# The Surgical Treatment of Carotid Body Tumor as Well as the Prevention and Management of Complications

**DOI:** 10.7759/cureus.51807

**Published:** 2024-01-07

**Authors:** Yasin Kilic, Izatullah Jalalzai, Ebubekir Sönmez, Bilgehan Erkut, Mansoor Jalalzai

**Affiliations:** 1 Cardiovascular Surgery, Ataturk University Hospital, Erzurum, TUR; 2 Surgery, Ataturk University Hospital, Erzurum, TUR

**Keywords:** carotid body tumor, carotid doppler, vascular mass, complications’, surgical treatment

## Abstract

The carotid body tumor (CBT) is a rare paraganglioma neoplasm that often occurs in the head and neck anatomical region. Carotid angiography continues to be widely regarded as the preferred diagnostic method for this particular malignancy. Surgical intervention has been widely acknowledged as the primary approach for managing CBTs. However, the resection of CBTs poses significant technical challenges due to its specific anatomical position. To mitigate the incidence of intraoperative and postoperative challenges, we have conducted a comprehensive review of both domestic and international literature to consolidate the surgical approach and strategies for preventing and managing complications associated with this particular tumor.

## Introduction and background

The carotid body tumor (CBT), also known as the glomus tumor, is the most prevalent paraganglioma found in the head and neck region. These tumors originate from the aggregation of chemoreceptor cells in the neck, which emerge from the neural crest region during embryogenesis. These tumors are considered rare, accounting for approximately 0.6% of head and neck neoplasms and roughly 0.03%. The increased incidence of CBTs has been associated with chronic hypoxemia resulting from chronic obstructive pulmonary disease and high altitudes [1]. Unilateral presentation is frequently observed, although those with a familial predisposition tend to exhibit bilateral manifestations in the majority of cases. The occurrence of this phenomenon is typically observed within the age range of 30 to 40 years [2]. The prevalence of the condition is greater in females than males. The majority of these disorders are considered benign, with malignant diseases comprising a range of 2% to 8% [3]. Only a small number of them have secretory capabilities. Research suggests that the prevalence of CBT may be influenced by genetic predisposition and prolonged exposure to hypoxic conditions in the surrounding environment. Estrogen is recognized as a significant contributing component in the acceleration of CBT development.

Painless cervical masses are commonly observed as initial manifestations of CBT. The progressive growth of the tumor can lead to the compression of adjacent tissues and infiltration of the skull base, posterior cranial nerves, and associated symptoms related to the sympathetic chain. These symptoms may include difficulties in swallowing, coughing during the ingestion of liquids, hoarseness, atrophy of the tongue muscles, Horner syndrome, and other related manifestations. In severe cases, compression of the vague nerve may also be observed. The presence of dizziness and Aspen syndrome, among other related conditions, is noted. There exist three primary indicators: the presence of the tumor within the carotid triangle, the superficial displacement of the carotid artery, and the separation of the internal and external carotid arteries. The presence of a tumor in close proximity to the bifurcation of the common carotid artery can be detected through palpation. This palpable mass exhibits pulsations and is capable of lateral movement but lacks vertical mobility. It is worth noting that patients with a surgical history may not exhibit this sign as prominently. Additionally, in some cases, a murmur may be perceptible either through touch or auditory means. The diagnosis of CBT mostly relies on imaging studies because of their close proximity to carotid arteries and nerves. This approach avoids the use of tiny needle aspiration and biopsy, as these procedures carry the risk of excessive bleeding and the development of pseudo-aneurysms [2].

## Review

**Figure 1:**
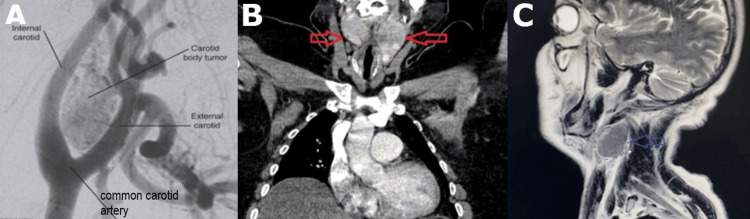
Radiologic diagnosis A) Angiographic view of CBT localized in carotid artery bifurcation; Rutherford's Vascular Surgery and Endovascular Therapy, 9th Edition, chapter 95. B) CT angiographic view of bilateral CBT (red arrows). Image Credits: Yasin kılıç; C) The salt and pepper appearance of CBT on MRI (blue arrow). Image credits: Izatullah Jalalzai. CBT, carotid body tumor; MRI, magnetic resonance imaging; CT, computed tomography

The utilization of color Doppler ultrasound, a straightforward and non-invasive diagnostic procedure, demonstrates a considerably elevated level of specificity and sensitivity in detecting CBT. The color-flow carotid duplex is considered to be the optimal screening test for CBTs [4]. Nevertheless, the limited application range of this technique is attributed to the occlusion of the mandible and the inadequate sight of small blood vessels. Currently, digital subtraction angiography (DSA) is widely regarded as the preferred method for preoperative diagnosis of CBT [5]. The most observable indication of CBT on DSA is the displacement of the internal and external carotid arteries, which manifests as a distinctive goblet-shaped alteration or "lyre sign." DSA has the potential to assess the degree of tumor infiltration in blood vessels preoperatively, assess cerebral collateral circulation using temporary balloon occlusion experiments, and predict the necessity of ligating the external carotid artery or sacrificing and reconstructing the internal carotid artery during surgical procedures. Furthermore, it has the potential to assist in preoperative planning: embolization, placement of stents, and various surgical procedures (Figure 1A). 

Research findings indicate that multi-slice spiral computed tomography (CT) has the potential to serve as a viable alternative to DSA for precise angiography. This is primarily due to its ability to not only effectively visualize the dimensions, configuration, and progression of tumors but also to delineate their spatial association with neighboring tissue structures. This is particularly significant in cases where tumors exhibit upward development and infiltrate the brain. Evidence of bone degradation can be observed in the lower region [6] (Figure 1B).

CBT has been observed to exhibit a distinct pattern akin to the appearance of "salt and pepper" on magnetic resonance imaging (MRI) scans. According to Guo Wei et al., MRI demonstrates a notable level of sensitivity, specificity, and accuracy in the assessment of carotid artery repair or resection [7] (Figure 1C).

**Figure 2:**
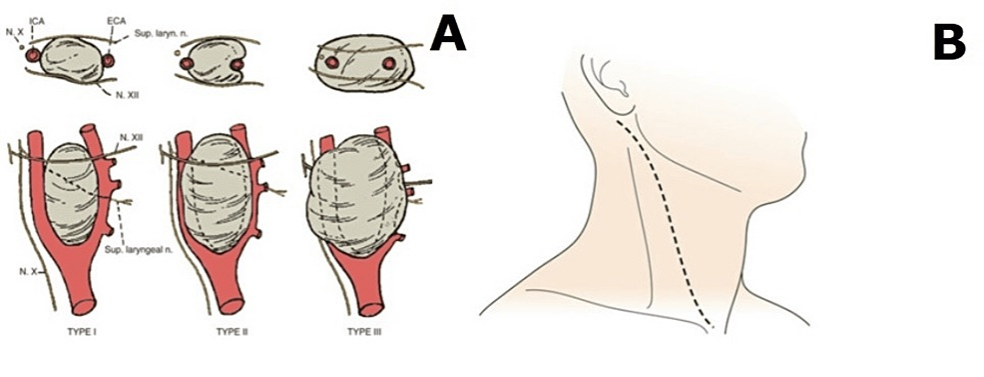
CBT Shamblin classification A) Shamblin classification of CBT; Rutherford's Vascular Surgery and Endovascular Therapy, 9th Edition, chapter 95. B) Surgical incision for CBT; Rutherford's Vascular Surgery and Endovascular Therapy, 9th Edition, chapter 95. CBT, carotid body tumor

Shamblin classified CBTs into three clinical kinds based on the extent of tumor involvement in the carotid artery (Figure 2A). There are three types of tumors in this context. Type I refers to a small tumor that can be readily separated from the carotid arteries without causing any damage. Type II, on the other hand, denotes a slightly larger tumor that partially encircles the carotid arteries. However, with meticulous dissection, it is possible to completely remove the tumor while preserving the integrity of the arteries. A subset of patients necessitates the implementation of bypass procedures and vascular reconstruction. Specifically, type III tumors are characterized by their substantial size, encompassing the carotid artery and bifurcation in their entirety and presenting evident symptoms and signs. In recent years, there has been a growing body of research indicating limitations in the Shamblin classification's ability to comprehensively assess neurological consequences and surgical complexity. As a response to this, Luna-Ortiz et al. put forth a refined alternative. The Shamblin classification, as proposed by Ma et al., posits that utilizing tumor texture as a basis for categorization can enhance the accuracy of predicting the challenges associated with achieving total tumor removal [8,9].

Due to its limited responsiveness to radiation and chemotherapy, surgical resection remains the primary therapeutic option for patients with favorable overall health conditions, while the efficacy of CBT tends to be gradual in nature. According to certain research, it is suggested that unilateral CBT demonstrates efficacy irrespective of the size of the tumor. Surgical intervention offers the dual benefits of tumor removal and alleviation of the tumor's impact on adjacent tissues, while also facilitating the acquisition of test specimens for pathological analysis. In contemporary practice, the anterior edge incision of the sternocleidomastoid muscle is frequently employed. In certain cases, this incision may be extended from the mastoid process's tip along the cervical crease to the neck's midline, allowing for a comprehensive visualization of the operative field (Figure 2B). In cases of large CBT, certain surgical procedures such as mandibular dislocation or mandibular amputation may be employed to achieve complete exposure [2,10]. For patients classified as Shamblin type III, who exhibit both intracranial and intracranial communication, a combination of cervical and suboccipital approaches can be considered a viable option.

Cervical block dissection represents a highly advantageous surgical approach, particularly well-suited for instances falling under Shamblin type I or those characterized by modest tumor sizes and inadequate blood supply. In cases where patients require vascular reconstruction surgery involving occlusion of the internal carotid artery, the implementation of a shunt can effectively safeguard cerebral blood supply, hence mitigating the occurrence of hemorrhage and neural impairment. Nevertheless, the utilization of arterial shunts has been associated with potential complications such as thrombosis or hemorrhage resulting from inadequate procedural execution, hence rendering their application a subject of ongoing debate [2]. Tumor excision, along with the closure of the internal carotid and common arteries, has demonstrated effective compensation for cerebral collateral circulation. Individuals experiencing challenges in vascular reconstruction may contemplate the utilization of this approach. It is important to note that significant problems, such as cerebral ischemia, have a higher likelihood of manifesting subsequent to the ligation of either the common carotid artery or the internal carotid artery. According to available reports, the prevalence of cerebral infarction ranges from 23% to 50%. Therefore, it is imperative to do a thorough preoperative assessment to ensure adequate patient care. The utilization of the external carotid artery in conjunction with tumor removal is considered appropriate for instances characterized by Shamblin types I and II, as well as those exhibiting a robust blood supply. The primary source of blood flow for CBT mostly derives from the ascending pharyngeal artery, which is a branch originating from the external carotid artery. Hence, the occlusion of the ascending pharyngeal artery situated cranially can result in a rapid decrease in tumor tension and size, hence facilitating improved visualization of the operative field [11]. In cases where the tumor reaches a significant size and there is recurrent damage to the external carotid artery during the separation procedure, it may be necessary to ligate the primary segment of the external carotid artery approximately 5 mm above its initial place of origin [12]. This intervention serves the purpose of minimizing bleeding during the surgical operation and enhancing the overall efficacy of the treatment. The surgical treatment of CBT aims to elucidate the potential disparity that may exist between the internal carotid artery and the tumor, with the objective of minimizing surgical duration and mitigating nerve impairment. It is important to note that the occlusion of the external carotid artery is not intended to be a permanent measure.

**Figure 3:**
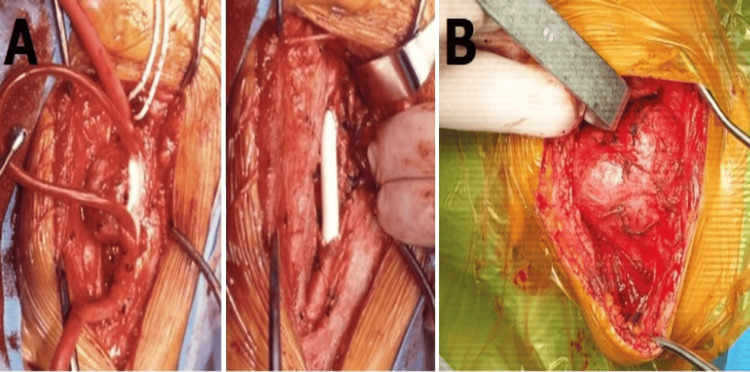
Intraoperative interpretation A) For tumors that involve the carotid artery bifurcation like Shamblin type III, the bifurcation and possibly the internal carotid artery must be removed from the tumor. A carotid shunt may be required during the reconstruction. The internal carotid artery can be re-vascularized with a prosthetic or autologous vein interposition graft (B); Rutherford's Vascular Surgery and Endovascular Therapy, 9th Edition, chapter 95. B) Shamblin type III CBT surrounding both internal and external carotid artery 360°. Image credits: Bilgehan Erkut CBT, carotid body tumor

Tumor resection and vascular reconstruction may be viable options for patients diagnosed with Shamblin types II and III. These types of tumors are characterized by their large size (diameter exceeding 5 cm) and abundant blood supply. Specifically, the tumor encases the internal carotid artery, with the upper boundary of the tumor positioned at least 1 cm away from the orifice of the carotid artery. Conduct vascular reconstruction. End-to-end anastomosis can be performed in cases when there is minimal strain observed following the excision of the affected segment, which measures less than 1 cm [13]. Carotid artery has the potential to be transplanted in situations where tension is present. The transplanted artery comprises several types, such as artificial grafts, autologous great saphenous vein, external carotid artery, and internal jugular vein (Figure 3A).

In the context of young patients and cost considerations, the utilization of the great saphenous vein as a primary alternative for carotid artery reconstruction is common. Certain studies suggest that the diversion of external carotid artery replacement offers superior outcomes in terms of caliber and reduction of cerebral ischemia duration. Several studies have proposed that arterial embolization prior to tumor resection may be a viable option for patients with limited tolerance to ischemia, such as those with bilateral CBT or extensive CBT reaching the skull base. Additionally, this approach may be suitable for patients with brain invasion and positive results on a temporary balloon occlusion test. The utilization of stent implantation has the potential to obviate the necessity for conventional vascular repair methods.

Histological evaluation alone is insufficient for distinguishing between benign and malignant cases of CBT. The presence of local lymph node metastasis or distant metastasis is a malignant trait, with local lymph node metastasis being the prevailing manifestation [14]. Given the challenges associated with preoperative and intraoperative pathological assessment of malignancy, it is advisable to do regular dissection for patients suspected of malignant tumor transformation or lymph node expansion during surgery. Additionally, it is important to prioritize the protection of the carotid artery to the greatest extent possible. The excision of the tumor margin, along with adjacent healthy soft tissue, is performed, followed by the inclusion of the tumor mass on the affected side. According to Davila et al., the authors held the belief that doing simultaneous lymph node dissection would not provide any further benefit in cases when there is no clinical suspicion of malignant tumors [15].

When bilateral CBTs are simultaneously removed, there is a notable increase in the rate and risk of complications. The endorsement of employing various operations is frequently recommended. Nevertheless, a comprehensive rationale for determining the initial resection site of the tumor has not yet been established. According to Ding et al., it is recommended to prioritize the treatment of the side with severe symptoms or a large tumor in patients with bilateral Shamblin types I or II CBTs, while simultaneously monitoring the contralateral side [16]. Conversely, for patients with bilateral types II or III CBT, it is advised to initially address the side with mild symptoms or a small tumor, followed by treatment of the side with more severe symptoms. The contralateral tumor exhibits strong adherence to adjacent blood vessels and nerves or demonstrates extensive invasion beyond the confines of the cranial region. Radiotherapy is an elective treatment option, and it is recommended that a minimum interval of six months be observed between two surgical procedures.

CBT is situated near the point of bifurcation of the carotid artery and exhibits a significant association with both arteries and nerves. The operation presents challenges and a significant occurrence of complications due to the unique characteristics of its anatomical structure (Figure 3B). In our department, we had to ligature the external carotid artery and reanastomose the end-to-end internal carotid artery after resection of Shamblin type III CBT.

The occurrence of bleeding is the prevailing intraoperative complication. Research findings indicate that the extent of blood loss during CBT procedures is mostly influenced by factors such as tumor size, disease progression in the patient, and the proximity of the tumor to the skull base [17]. In cases where tumors exhibit larger diameters or are situated at higher positions, the implementation of preoperative embolization (within a span of 1 to 3 days) targeting the branches of the external carotid artery and vessels supplying the tumor can effectively mitigate intraoperative bleeding and minimize the need for carotid artery reconstruction [18]. However, it is important to note that this approach carries the potential risk of reflux embolization of the embolic material. The use of a precise delineation between tumors and blood vessels during surgical procedures, coupled with the utilization of bipolar electrocautery, has been shown to effectively mitigate vascular damage and minimize bleeding that occurs intraoperatively [2]. Research has indicated that the act of blunt separation has been associated with the development of pseudoaneurysms, as evidenced by multiple studies [2]. According to the literature, in cases when patients have considerable bleeding during surgery and develop severe anemia post-surgical, it may be appropriate to seek symptomatic treatment through blood transfusion [19].

The occurrence of ischemic stroke during CBT surgery is considered a significant complication, with a documented incidence rate of approximately 7%. Several studies have reported a correlation between the incidence of ischemic stroke and the manifestation of symptoms associated with carotid artery compression prior to surgical intervention, as well as the presence of the tumor mass. The risk of stroke is more related to the Shamblin categorization types II and III, the prolonged duration of intraoperative blockage of the internal carotid artery, the reconstruction of the internal carotid artery, and the presence of asymptomatic plaques in the impacted carotid artery. Hence, it is imperative to thoroughly assess and comprehend the state of the Willis polygon before surgical intervention and precisely quantify the brain's compensatory blood circulation. Following direct repair or patch repair procedures, it is customary to administer either single or double antiplatelet therapy to patients. Individuals who undergo the surgical repair of autologous blood vessels and artificial vascular grafts are typically prescribed anticoagulant therapy and long-term antiplatelet therapy for a minimum duration of six months. This treatment regimen is implemented with the aim of preventing the occurrence of thrombosis.

The primary complication following cranial base tumor treatment is cranial nerve injury, with the most often affected nerves being the vagus nerve and the hypoglossal nerve. Despite the advancements in surgical technology, studies have reported that the incidence of cranial nerve injury following cranial base CBT resection remains high, ranging from 20% to 44% [18]. Some studies suggest that several factors contribute to this high risk, including inadequate preoperative evaluation, the presence of large- and medium-sized tumors, Shamblin types II and III tumors, and prior invasive neck operations [18]. Hence, it is imperative to prioritize early diagnosis, thorough examination, and prompt resection as the primary strategies for preventing cranial nerve damage. Additionally, ensuring comprehensive exposure of the operative region is crucial in minimizing the risk of cranial nerve damage [20]. Hence, it is imperative for patients who have received a clinical diagnosis of CBT to exercise caution in undergoing invasive neck surgeries prior to their treatment. Additionally, it is crucial to thoroughly assess the interplay between the tumor type and the adjacent anatomical structures prior to surgical intervention. Furthermore, patients should possess a comprehensive understanding of the morphology of the neck nerves. It is recommended to employ bipolar electrocautery in close proximity to blood vessels and nerves whenever feasible. In the process of tumor excision, it is advantageous to initiate separation from the distal portion of the tumor. This approach facilitates the complete exposure of the cranial nerves while minimizing the need for clamping and excessive retraction [21]. It is imperative to emphasize the identification and safeguarding of nerves. In certain cases, following surgical procedures, medicines such as dexamethasone and mannitol may be used to mitigate the swelling of nerves and cerebral edema resulting from brain reperfusion after diversion.

Tracheal intubation and tracheotomy procedures are employed in cases where patients exhibit vague nerve injury and vocal cord paralysis, based on the complex surgery of the CBT. Baroreceptor failure syndrome is a medical condition characterized by the loss of blood pressure regulation due to the bilateral removal of carotid artery baroreceptors during bilateral CBT resection. The occurrence of bilateral CBT is estimated to be between 5% and 10% in sporadic occurrences, whereas it constitutes approximately 30% of familial CBT. According to certain research, it has been suggested that baroreceptor failure syndrome can exhibit four distinct manifestations, namely hypertensive crises, abrupt variations in blood pressure, increased heart rate, and heightened sensitivity of the vague nerve. These manifestations may potentially be accompanied by symptoms such as anxiety, headache, and other forms of discomfort. Presently, the focus of therapy lies in the prevention of problems arising from hemodynamic instability, rather than the restoration of pressure receptor function. Clonidine has been found to effectively decrease the release of norepinephrine from sympathetic neurons, which is associated with the sensitivity of the baroreceptor reflex. This reduction in norepinephrine release contributes to the alleviation of hypertension and tachycardia. Consequently, clonidine is currently considered the preferred pharmacological intervention for managing baroreceptor failure syndrome. In the management of acute episodes of elevated blood pressure, pharmacological interventions such as sodium nitroprusside, phentolamine, labetalol, and other antihypertensive medications are commonly administered to alleviate symptoms. The primary objective of these treatments is typically to sustain systolic blood pressure below 180 mm Hg. In addition to its primary use, clonidine may be employed in conjunction with β-blockers and benzodiazepines for the management of anxiety [22].

It is important to acknowledge further complexities that may arise during functional CBT surgical interventions, including intraoperative malignant hypertension and postoperative persistent hypotension. Hence, it is imperative to administer α-blockers and β-blockers to patients of this nature prior to undergoing surgery. In order to regulate blood pressure during surgical procedures, it is recommended to administer short-acting antihypertensive medications, such as sodium nitrate and nitroglycerin, as opposed to long-acting alternatives. It is recommended to discontinue the administration of vasodilator medications a few minutes prior to the conclusion of tumor excision. In the event of postoperative hypotension, treatment options may include the use of dopamine and adrenocortical hormones [23-25].

## Conclusions

CBTs are rare, benign neuroendocrine growths in the neck, often presenting as inconspicuous masses with symptoms like cough and dysphagia. Diagnostic tools include Doppler ultrasound and CT, with MRI being considered better due to its radiation-free nature and ability to outline soft tissue extension. The only proven treatment for CBTs is complete surgical removal. Preoperative imaging techniques like CT or MR angiography are crucial for providing detailed anatomical information. Preoperative embolization can minimize intraoperative hemorrhage and reduce the risk of cranial nerve injury. Advances in surgical technology and interdisciplinary discussion models offer the potential for more scientific treatment plans, reducing intraoperative and postoperative complications.
